# Discussing the Drawbacks of the Implementation of Access and Benefit Sharing of the Nagoya Protocol Following the COVID-19 Pandemic

**DOI:** 10.3389/fpubh.2021.639581

**Published:** 2021-12-10

**Authors:** Sally Mueni Katee, Christian Keambou Tiambo

**Affiliations:** Centre for Tropical Livestock Genetics and Health (CTLGH), International Livestock Research Institute (ILRI), Nairobi, Kenya

**Keywords:** COVID-19 pandemic, Nagoya protocol, ABS, genetic resources, DSI

## Introduction

“When we think of the major threats to our national security, the first to come to mind are nuclear proliferation, rogue states, and global terrorism. But another kind of threat lurks beyond our shores, one from nature, not humans - an avian flu pandemic” (1). In 2005, Barack Obama and Richard Lugar identified and vocalized the need for a permanent framework that would be used in reducing the spread of infectious diseases. In the realm of infectious diseases, a pandemic is always the worst-case scenario. With over 200,000,000 globally confirmed cases and over 4 million deaths, COVID-19 is the reason behind the turbulent start of a new decade; COVID-19 also marks the beginning of a new era where nothing in the world will ever be the same (2). The pandemic has induced several vast changes that have resulted in the adaptation of a new way of life. We have experienced unprecedented social and economic disruptions that have pointed out the significance of rapid pandemic response and recovery mechanisms.

Both the samples and the digital sequence information (DSI) of the SARS-CoV-2 that causes COVID-19 were collected and called to be part of the operationalization of fair and equitable benefit sharing, as recognized by the Convention for Biological Diversity and Nagoya Protocol (3). The rapid sharing of these samples and their DSI have been pivotal to the discovery of research work in diagnostic, therapeutics, and COVID-19 vaccine development. Forty global and regional civil society organizations, 228 national organizations, and 124 individuals from 77 countries expressed the need to the UN Secretary-General and the WHO Director-General to facilitate a “coordinated global research roadmap” to rapidly find a solution to COVID-19. Countries, organizations, institutes, conglomerates, and scientists have all come together to fight the battle against this modern-day pandemic.

The first genetic sequence data for SARS-CoV-2 was generated by the Chinese Center for Disease Control and Prevention in a record time of 16 days after the Wuhan outbreak in January 2020 and a week after Beijing's outbreak in June 2020 (4). The same authors revealed that data was freely and rapidly shared with the Global Initiative of Sharing All Influenza Data. Similar to influenza, SARS-CoV-2 has mutated and already spread around the world. Keeping in mind the provisions and legally binding obligations arising from the Nagoya Protocol, an issue of concern is whether China would have been able to rapidly share the SARS-CoV-2 genetic sequences had it followed the requisite procedures. COVID-19 serves as a reminder that frameworks governing the use of genetic resources should avoid impeding the research community, especially in emergencies. However, these laws should be structured in a way that does not undermine the sovereignty of countries over their genetic resources, be they pathogens of other forms of biological material.

In 2020, the pandemic resulted in various travel bans and restrictions. It also wreaked havoc on economic activity, resulting in what seems to be the present-day “stock-market crash” (5). Multiple businesses have been forced to close their doors, turning their backs on their employees. Furthermore, because of nationwide lockdowns, the non-essential workers are left confined to their homes. Some countries have taken different approaches varying from total confinement (early days in China), partial lockdown (Kenya), and to more flexible methods (Sweden).

## Brief Legislative Framework Background

**Figure 1:**
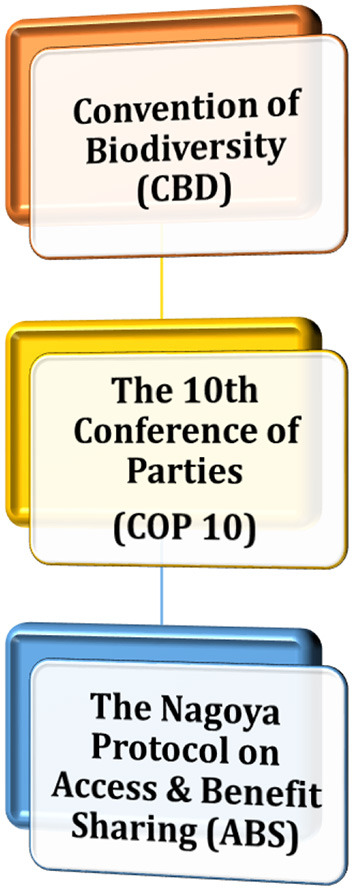
Legislative framework background.

As sequentially illustrated by Figure 1, the tenth meeting of the Conference of the Parties (COP10) to the Convention on Biological Diversity (CBD) was held in Nagoya, Aichi Prefecture on the October 18, 2010 (6). The 10-day conference had over 13,000 participants from different parties to the convention, relevant international, and nongovernmental organizations (6). A key priority for this meeting was the initiative to support the compilation of national strategies on biological diversity aimed at assisting countries in the development of capacity building regarding access and benefit-sharing (ABS) related to genetic resources. COP10 adopted the Nagoya Protocol on ABS and the New Strategic Plan of the CBD (the “Aichi Target”) from 2011 onward (6). The protocol officially came into force in 2014 (7).

The Nagoya Protocol is an internationally binding treaty, and the third component of the protocol highlights the “fair and equitable sharing of the benefits arising out of the utilization of genetic resources” (7). Focusing primarily on the third objective of the CBD, the Nagoya Protocol defined the “rules of the game” outlining the requisite sharing of genetic resources between countries (8). It determines that the genetic resources in principle are owned by the country where they have been found or by whoever the government decides to grant ownership to.

The Nagoya Protocol enforces the concept of state sovereignty by giving countries the ability to determine, control, and monitor the use of biological material accessed within their territory (7). This is guaranteed by way of Material Transfer Agreements (MTAs), Prior Informed Consent (PIC), and Mutually Agreed Terms (MAT) between the Provider and the User. The aforementioned is catered for in Article 6 of the Protocol. Before any transfer of genetic material occurs, a consortium must be in place. The users of the genetic material must comply with the requisite procedures and domestic laws of the providing country. All parties must agree on the terms before the transfer of the material. These negotiations, procedures, and technicalities often consume a lot of time as every party is trying to ensure the transaction protects their rights and interests unequivocally.

**Figure 2:**
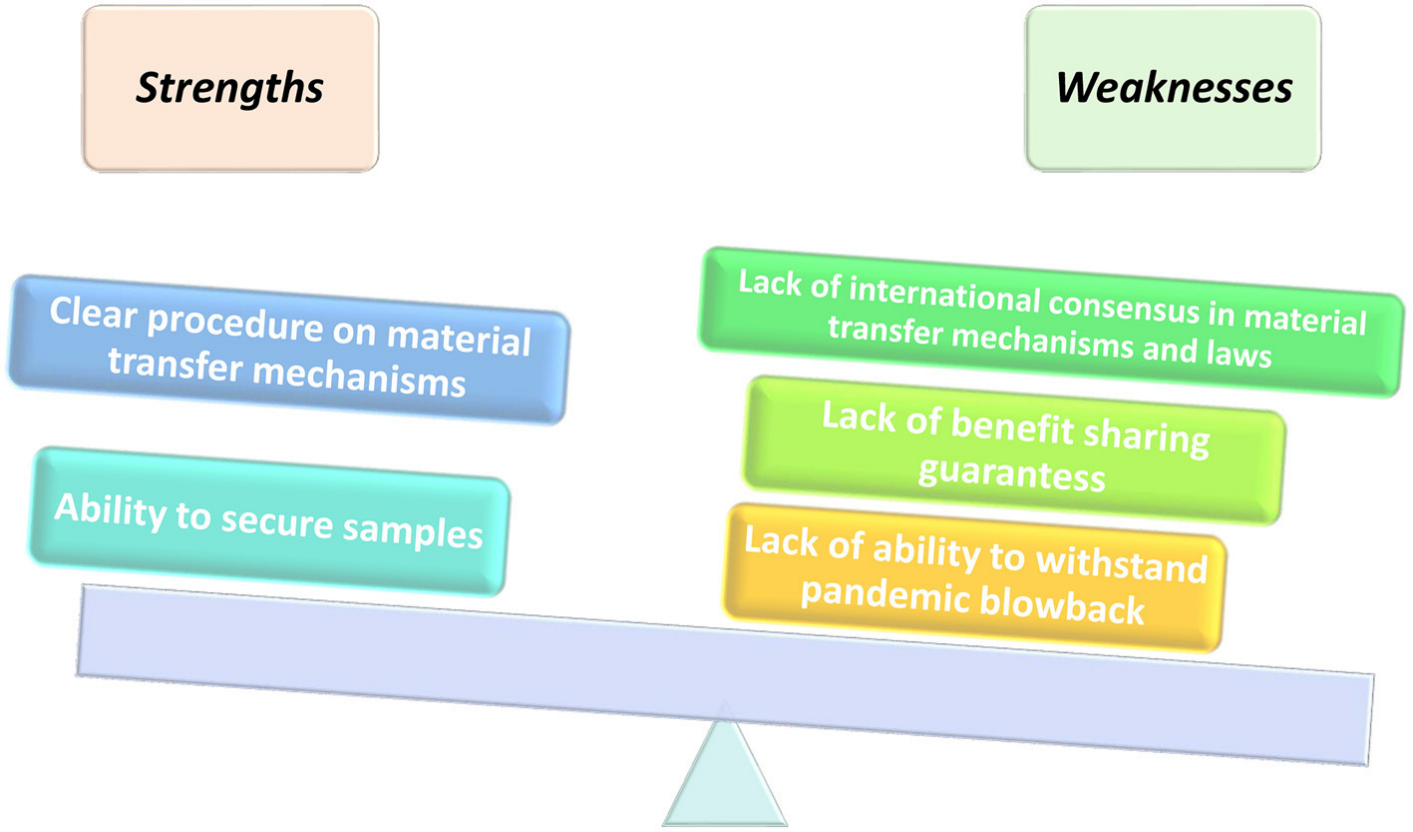
Strengths and flaws of the current PIP framework.

Despite the strengths and flaws in its implementation framework as illustrated in Figure 2, Article 4 (2) of the Nagoya Protocol provides that “Nothing in this Protocol shall prevent the Parties from developing and implementing other relevant international agreements, including other specialized access and benefit-sharing agreements, provided that they are supportive of and do not run counter to the objectives of the Convention and this Protocol.” As a result of the pandemic, the COVID-19 samples and genetic sequence information were called to be part of the operationalization of fair and equitable benefits sharing, as recognized by the Convention for Biological Diversity (CBD) and Nagoya Protocol. The WHO has specific frameworks in place that regulate and pandemics related to human health. Even though access and benefit sharing (ABS) is primarily catered for in the Nagoya Protocol, it leaves many gray areas on the standard mode of operation during a pandemic. There is a need to unpack the Nagoya Protocol in its entirety to understand the appropriate implementation during a pandemic.

Keeping in mind the working mechanism of the Nagoya Protocol, there is a need to understand the scope of genetic resources as this highlights the importance of this discussion during a worldwide pandemic. The term “Genetic Resources” refers to anything that contains genetic material (DNA, RNA) like plants, animals, microbes, and human beings (8). The Convention for Biological Diversity (CBD) - Nagoya Protocol (NP) regulates all of these except the human genome. However, in its definition of genetic resources, the terms used in the Nagoya Protocol have collectively been construed to include microbes (bacteria, parasites, viruses, and fungi) that may infect humans, animals, and plants. As a result, the use of pathogens for public health purposes is subject to the ABS requirements and procedures of individual countries (8). This automatically means that the country from which a virus sample was isolated has the sovereign authority to determine how and by whom that sample is utilized. This potentially affects all international sharing of any and such materials, even though they are to be used for public health purposes.

## The Nagoya Protocol Implementation and Public Health Implications

The Director-General of the International Federation of Pharmaceutical Manufacturers (IFPMA) is of the view that the Nagoya Protocol potentially hinders research collaboration specifically in instances where the development of a new vaccine or treatment for a new virus or other pathogen is of utmost urgency (9). He further argues that had China followed the requirements of the Nagoya Protocol following the discovery of SARS-CoV-2, it could have embarked on discussions with each country, one by one, on how to share the sequence of this pathogen. This would have initiated the process of bilateral negotiations with the governments of all the interested countries. Subsequently, this would mean that the parties willing to access and use these genetic resources for outbreak-related research and response would have had to negotiate conditions bilaterally with governments for each material needed (8). Hypothetically speaking, in the event of an international scientific consortium that would require diagnostic testing for a new pathogen, the collation of samples from all affected countries would be imperative to this exercise and would require approval from all the member states to access and use the various samples. This is a very daunting scenario, especially during a public health crisis where time is very important. There are enormous demands for rapid access to information about this new virus, the patients and communities affected, and the response, but equally crucial is the need to ensure that this data is reliable, accurate, and independently scrutinized (9). As fate would have it, we are currently living in a time where we need not hypothesize these issues anymore. COVID-19 is a public health crisis, therefore it is a concern for all of humanity. Subsequently, this raises the need for an assessment on how pandemic preparedness and response as a global community has previously, currently, and potentially been affected by the current implementation of the Nagoya protocol.

It took slightly over a week for WHO to confirm the existence of the new coronavirus and for the Chinese scientists to publish its genetic sequence after the outbreak's first report. This efficiency and swiftness are the first of their kind. The rapidity of this information dispensation still remains unprecedented. This is only possible because the WHO's policy on ethical issues and outbreak management mandates the rapid sharing of data during an unfolding health emergency, as this aids in the identification of etiological factors, prediction of disease spread, evaluation of existing and novel treatments, symptomatic care and prevention measures, and lastly the guidance on the deployment of limited resources (9). Although this policy advances the efforts of the public health sector during an unfolding health emergency, it constitutes a breach of the Nagoya Protocol. There is a need to evaluate the magnitude of the effects occasioned on the public health sector as a result of the current implementation of the Nagoya Protocol, especially about existing and emerging infectious pathogens that require global research consortia to save lives.

As mentioned in the preceding sections of this study, the Nagoya Protocol enforces the concept of state sovereignty, giving countries the ability to set out conditions that determine the way their genetic material will be transferred and used. Viral sovereignty, as defined by Inkstone (10), continues to be an issue that has inadvertent effects on public health. In addition, political dissent always seems to push this agenda further. In 2007, Indonesia refused to give WHO their samples of an H5N1 influenza strain from an outbreak in the country until it was guaranteed fair access to any vaccines created from that material (10). Similarly, in 2018 and without any explanation, China withheld laboratory samples of the H7N9 bird flu despite repeated requests from the United States and the United Kingdom to share the material (11). In 2020, the United States Intelligence has accused Beijing of concealing information about the COVID-19 outbreak, claims that the Chinese authorities have rejected (12). Several other elements challenge the sovereignty claims that a country may lay on a virus. The transmissibility nature of the virus and its effect on the human population are some of the elements to consider.

The crux of the issue in this study is that time and time again, the global community has failed to reach a consensus on the scope of the exact obligations that States have to share genetic sequence information relating to pathogens, more so amid a worldwide pandemic. The ownership of pathogens and related information emerging in different states is part of a long-standing debate. A discussion that touches an exploitative colonial nerve suggests that wealthy countries still plunder the natural resources and biodiversity of poorer nations and are actively profiting from it (10). Furthermore, there are dissenting opinions about whether Digital Sequencing Information (DSI) is covered in the Nagoya protocol.

The US, European Union, and Japan have consistently argued that the NP applies only to tangible biological materials; however, many other countries including Brazil, Ethiopia, India, and Malaysia assert that the protocol also applies to information from genetic resources, including DSI (13). In implementing their legislation for the protocol, these countries and others are applying ABS requirements to DSI from pathogens. Scientists are concerned that the soured political atmosphere combined with loopholes in existing international frameworks could impede the sharing of genetic data and virus samples in the future.

One thing that is clear is the fact that the Nagoya Protocol serves as one of the existing international frameworks for access and benefit-sharing of genetic resources. The different interpretation of the protocol and approaches for the implementation of different domestic legislation on the same has often been the genesis for the claims of sovereignty for isolated pathogens. The Nagoya Protocol provides that some of its measures may be implemented through policy, legislative, and administrative instruments which bring about non-uniformity. None of the provisions of the Nagoya Protocol so far has been the subject of a judicial interpretation. It is important to take note of the dissenting views and how they affect the intended interpretation of the Nagoya protocol.

Consequently, the WHO has tried to resolve the issue with the pandemic influenza preparedness (PIP) framework that was adopted in 2011 (10). These rules affirmed state sovereignty as a legal norm and imposed no direct legal ramifications for not sharing influenza viruses with the WHO. Further, WHO's International Health Regulations mandate the member states to notify the organization with all relevant information that would result in a public health emergency of international concern (13). At the same time, the rules having been formulated in an international law context are a form of international cooperation. This debate has reemerged in the context of COVID-19, particularly on state obligations to inform the world when a pandemic outbreak occurs, and the WHO's responsibility to declare a pandemic. Unfortunately, these rules do not classify genetic sequence data or physical pathogen samples as health information, and it is unclear whether these regulations and the PIP framework apply to COVID-19.

The COVID-19 death toll has displayed different patterns in various parts of the globe, and this has raised the question of whether countries can claim ownership of pathogens that have emerged within their borders and if so, how do we guarantee sharing of benefits? As well as the costs of losses occasioned by the pathogen? This is a question which was asked in the past, not only in the context of H5N1 but also in biocontrol of plant pests with countries that host the natural predator to a pest asking whether they will share in the benefits arising from the eradication of the pest in the receiving country (14). The normal response is whether they would be willing in the first place, to share in the losses occasioned by the pest. These gaps and flaws in the implementation of the Nagoya Protocol have very far-reaching impacts on global public health. Impacts include impediment and unnecessary delay in international research collaborations, pathogen sample sharing, infectious disease research, pandemic and epidemic preparedness and response, medical countermeasure development efforts, and investor interest in vaccine development.

These effects on public health are visible, extreme, and very real. Even though the World Health Assembly Report on the Public Health implications of implementation of the Nagoya Protocol posited that the protocol actively provides an opportunity to advance public health, it failed to consider the other significant risks occasioned by the treaty (15). The current implementation of the protocol has been at the origin of significant delays in sharing influenza viruses' information, including from national influenza centers in Southeast Asia and South America with a long-standing record of timely sharing as required under the terms of reference in the Global Influenza Surveillance and Response System (GISRS) (15). Those national influenza centers found themselves having to delay the sharing of influenza viruses due to conflict with national legislation on ABS arising from the recent implementation of the Nagoya Protocol and consequently missed the timing for the seasonal vaccine composition meeting (16).

A similar situation occurred in Europe as well where the WHO Collaborating Centers of GISRS experienced a delay of 3 months before a candidate vaccine virus, falling under France's Nagoya Protocol legislation, could be shipped to manufacturers. In another case, in Switzerland, there was a delay of 3 weeks in the ability to use a WHO-recommended candidate vaccine virus for manufacturing due to a lack of clarity of the consent process to be followed and who the “user” of the strain was; furthermore, it was not clear whether seasonal influenza fell under the scope of the Swiss ABS legislation or not (16).

Delays in virus sharing often harm the vaccine development procedure as the quality of the vaccine and its supply are actively compromised. These delays further affect the timeliness and comprehensiveness of the entire procedure. The ABS principles should attempt to find a balance between protecting the interests of providers and users whilst aiding and enhancing public health and pandemic preparedness and response. It is imperative to public health that the present ABS mechanisms be amended to encompass and ensure global systems that guarantee global benefit sharing. The global community must begin to ask itself as to what can be done to effectively protect public health equities in the context of the Nagoya Protocol and national-level ABS implementation?

The principle that countries should equitably share benefits arising from the utilization of genetic resources in their jurisdictions is no new feat as it is catered for in the UN Convention on the Law of the Sea (adopted in 1982) and the CBD (adopted in 1992). The Nagoya Protocol and the WHO, PIP framework (WHO–PIP) are just the latest additions to the expression of this principle. National ABS legislations therefore can be viewed as a basic expression of the “general principles of law recognized by civilized nations” (art. 38 of the statutes of the ICJ).

In 1948, the WHO was founded and trusted to establish the canons that are currently in place for global health. Enshrined in the WHO constitution as one of the main functions of the organization is the stimulation and advancement of the work to eradicate epidemic, endemic, and other diseases. In addition, the UN body's mandate is further strengthened by its capacity to promote and establish guidelines on public health, preventive care, clinical medicine, ethical research, and ensuring that emerging technologies improve worldwide safety and well-being (17). WHO has successfully developed a wide array of guidelines and principles that have previously and are still being used to promote global health.

Beneficence, reciprocity, and solidarity are often terms that should be considered when taking a glance at the principles used to manage ethical issues during infectious disease outbreaks. From the onset, different obligations arise for both governments and the international community in the event of infectious disease outbreaks. Governments play a critical role in preventing and responding to infectious disease outbreaks by improving the social and environmental conditions, facilitating the provision of well-functioning and accessible health systems, as well as engaging in public health surveillance and prevention activities (18).

States have an ethical obligation to ensure that they are equipped with the long-term capacity of the necessary systems required to carry out effective epidemic prevention and response. However, these are not the only obligations that countries have, they extend beyond their borders. All countries must carry out their responsibilities under the International Health Regulations (IHR) to participate in the global surveillance efforts truthfully and transparently. This includes providing prompt notification of events that may constitute a public health emergency of international concern, regardless of any negative consequences that may be associated with the notification. Negative consequences would include issues such as a potential reduction in trade or tourism (18).

Referring to the preceding sections of this study, it was mentioned briefly that global pandemic response has continually faced challenges, more so at the pathogen sample sharing stage. This is an issue that was brought to the worldview in 2007. Spearheaded by Indonesia, matters regarding ABS were for the first time placed in the limelight. A further look at the state of affairs in this Southeast Asian Republic unearthed some rather appalling discoveries as Siti Fadilah Supari, the Minister of Health at the time came forward and announced that they would immediately suspend the sharing of virus samples with the WHO Collaborating Centers. This was attributed to the fact that Indonesia at that time was severely affected by the highly pathogenic H5N1 virus. Furthermore, it was brought to their attention that the virus samples they had shared with the collaborating centers had been used for vaccine development without their consent and were subsequently being offered to Indonesia by an Australian drug company at $20 a dose (19). This was a rather unfortunate and unfair twist of events. The Indonesian population at the time stood at over 200 million, the amount of money required to purchase the drug from the Australian company was unfathomable if not absurd. It was clear that the “provider” countries were being exploited although they shared their samples in good faith.

At the time, the situation dictated that the affected countries would send potentially pandemic avian flu virus samples to certain national laboratories designated as the collaboration centers. These laboratories were in developed countries and would sequence the virus thereafter developing candidate vaccine strains (20). Unfortunately, in violation of the WHO guidelines, they sent the viruses to the commercial sector for vaccine development, without the consent of the providing countries. Worse still, the vaccines developed by the private sector, using the samples accessed from the Global Influenza Surveillance Network (GISN) were unavailable and/or not affordable to developing countries. It also soon became apparent that the GISN's operations were inconsistent with the principles and provisions of the CBD that required PIC and MAT to kick off the material transfer process. Based on these controversies, these issues were discussed at the 60th World Health Assembly, kicking off tense negotiations that lasted 4 years and eventually led to the adoption of the PIP framework.

The year 2011 saw the members of the WHO adopt a ground-breaking agreement: the PIP framework. The WHO–PIP framework for the first time provided a link between access to pathogens and the fair and equitable sharing of benefits arising from their use (20). The PIP framework aimed at building on the legal principles encompassed in the CBD, recognizing the sovereign right of states over their biological resources. Furthermore, the PIP framework recognized that the members of the WHO have a commitment to virus-sharing and benefit-sharing on an “equal footing,” as they are “equally important parts of the collective action for global public health.”

The main objective of the PIP framework is to improve pandemic influenza preparedness and response. It also aims at strengthening the protection against pandemic influenza by improving the Global Influenza Surveillance and Response System (GISRS), thus resulting in a fair, transparent, equitable, efficient, and effective system. Over the years, the PIP framework has largely been considered to be a success story. Sharing of seasonal influenza viruses and influenza viruses with human pandemic potential (IVPP) is governed by two different but mutually reinforcing and supportive regimes (21). These are the GISRS and the PIP framework.

Under these regimes, National Influenza Centers (NIC) are designated by the health ministry of the country concerned and are recognized by WHO. The designation requires formally agreeing to comply with the GISRS seasonal influenza terms of reference (TORs) and the PIP framework, under which NICs agree, inter alia, to share influenza virus samples with other GISRS and non-GISRS laboratories. It is important to take note of the fact that the IVPP framework does not extend to seasonal influenza viruses or any other pathogens (20).

The framework subjects all transfers of the IVPP among the WHO–GISRS laboratories and with entities outside the GISRS system to the standard MTAs (SMTAs) and commits all recipients of PIP biological material to benefit-sharing. In addition, the framework also puts in place a transparent traceability mechanism, the influenza virus tracking mechanism, which tracks real-time the movement of PIP biological material into, within, and out of the WHO–GISRS.

Five years after the implementation of the PIP framework, an expert review commended the framework, referring to it as an “essential instrument” for pandemic influenza preparedness. The report further posited that the implementation has led to greater confidence and predictability in the global capacity to respond to an influenza pandemic (20). True as this may be, the PIP framework has still left a lot of stones unturned. The PIP framework presents a different set of both continuing and developing challenges, specifically those relating to other pathogens shared within the network of the WHO.

In the wake of continuous technological developments, the issue of DSI still presents itself as a challenge for the PIP framework. It is no secret that thanks to the combined efforts of scientists across the globe pathogens can be developed, modified, and generated from DSI. Matters relating to DSI in both the NP and the PIP framework continue to be an area that lacks global consensus. These issues ought to be handled with utmost importance and urgency mainly because most of the research conducted as a result of pathogen isolation yields benefits that the initial providers are most times unable to access. Genetic material and the DSI resulting from the same material ought to be viewed in equal light by all the frameworks involved.

There is a need to actively enforce a balanced data-sharing ABS model for other pathogens. The SARS and MERS outbreaks were full of controversies as the scientists tasked with fighting the outbreak applied for virus genome patents (20). Furthermore, pathogen samples were actively being shared without the consent of the provider. These controversies are symptomatic of the inequities and bias prevailing in global health governance. The WHO is uniquely positioned with the capabilities and the resources to facilitate pandemic preparedness at the national and international levels. In addition, they are more than capable of developing benefit-sharing structures for other pathogens shared in situations of emergencies. There is a need to develop international rules governing the use of pathogens and DSI, especially those establishing fair and equitable benefit-sharing consistent with the objectives and provisions of the CBD and the Nagoya Protocol (20). It is also imperative to the global community that this process involves all stakeholders.

The lack of international rules governing access to pathogens, fair and equitable benefit-sharing is a major deficiency. This brings to life the potential risk of the reoccurrence of controversies seen during the SARS, MERS, and avian flu. Unfortunately, this would result in the erosion of trust and the weakening of pandemic preparedness and response. Despite the current outbreak of COVID-19, these controversies are still with us.

Amidst the quest for COVID-19 treatment, Dr. Tedros Adhanom Ghebreyesus, the WHO Director-General, has come forward to support the idea of creating a voluntary pool to collect patent rights, regulatory test data, and other information that could be shared for developing drugs, vaccines, and diagnostics (22). An idea that has not received the warmest welcome as pharmaceutical companies across the world have openly expressed their resistance to this idea (23). This idea is premised on the fact that COVID-19 medical products may not be accessible for poorer populations. By establishing a voluntary mechanism under the auspices of the WHO, the goal is to establish a pathway that will attract numerous governments, as well as industry, universities, and nonprofit organizations.

The proposal for a patent pool is modeled around the medicines patent pool and was initially proposed by Costa Rica. It has other proponents (Netherlands) and opponents (US, UK, and others) with each side having its arguments. This pool could potentially provide a system of enabling deployment (access) of pharmaceutical products to large masses rapidly, as opposed to if the patents were under the control of one or fewer entities. Speaking at a forum organized by IFPMA, Pascal Soriot, the Chief Executive at AstraZeneca argued that intellectual property (IP) is a fundamental part of the pharmaceutical industry, and the potential lack of IP protection extracts all innovation incentives (22). He further added that the present issue of importance is the voluntary provision of products, at no profit, in the time of pandemic crisis.

The lack of a foundational balanced model of reciprocity for global public health that could be applied to other pathogens will always create a reoccurrence of the aforementioned controversies. Consequently, this frustrates all efforts to move forward with global health. There is a need to objectively look at the inconsistencies at hand and deal with them once and for all to avoid inequities in global health and overall inefficiency in pandemic preparedness and response.

## An Access and Benefit-Sharing Model for Other Pathogens

It is evident that there is a need for the global community to kick off the discussion on the regulation and management of other pathogens. COVID-19 has been able to illuminate the flaws of the existing pathogen-specific ABS instrument. It has also been able to identify the fact that the documents in development should attempt to address those flaws sufficiently. However, to identify the specific problems that the proposed ABS sharing model should address, we must discuss the flaws of the existing pathogen-specific ABS instrument, which is the PIP framework.

The Milbank Quarterly Journal in 2019 revealed that during an influenza pandemic, the PIP framework is likely to secure access to necessary virus samples but highly unlikely to secure the promised benefits for countries in need (24). This established that in practice the PIP framework only upholds one side of the access and benefit-sharing bargain. This often leaves countries unsettled because if the framework is unable to secure promised benefits like vaccines and antivirals, then they may feel they are better positioned to protect their populations from an influenza pandemic by conducting the access and benefit-sharing transaction outside the remit of the multilateral PIP framework. Unfortunately, this results in the direct transaction between the provider and the potential users of the resources, and the position assumed by the WHO as an intermediary is rendered redundant. This kind of scenario has ripple effects that would potentially result in interference of the entire global surveillance system that has been vital to monitoring and responding to the threat posed by influenza.

In addition, a further look at the PIP framework suggests that during a pandemic, the framework would not be able to withstand the blowback, yet this is the very basis of the document's creation. One of the main inconsistencies with the PIP framework is the fact that the SMTA does not create any directly binding agreements between the member states and third-party recipients of influenza viruses. In the lead-up to and during a pandemic, the SMTA1 secures access to influenza viruses for the WHO and the SMTA2 secures access for commercial users of virus samples. However, the SMTA2 may be ineffective in securing tangible benefits for the sovereign providers of those materials. An issue for consideration is whether the PIP framework through the SMTA1 and SMTA2 creates a multilateral system of access and sharing of benefits, and if so, what is the scope of this system? In addition, how do we ensure that this system's operational outlook delivers sufficient and tangible results?

In a bid to enhance engagement among stakeholders, Manheim (13) stated the need to actively identify and provide examples of monetary or non-monetary benefits to the global public health system. Specifically, the examples are facilitated by international sharing of pathogens, biospecimens, pathogen genetic sequence data, and/or relevant metadata. Manheim (13) further expressed the need to identify other pathogen-specific issues and examples that could affect global pandemic preparedness and response or efforts to combat seasonal outbreaks. It is also important for us to identify the non-ABS challenges and barriers to sharing pathogens internationally or those that might merit additional attention or analysis due to the significant implications they would have on global pandemic or epidemic preparedness and response efforts. More research is necessary to examine the possible course of action for a working ABS model that can deliver for other pathogens, especially under the pressure of a pandemic.

## Recommendations and Conclusions

The One Health approach has often been mentioned when considering the alternative avenues for other pathogen-specific ABS models. Comprehensive research attempting to address the present-day challenges has never been greater. Sharing of information, data, and interdisciplinary collaboration are at an all-time high. The One Health approach to research ensures that human, animal, and environmental health questions are evaluated in an integrated and holistic manner. This aims to provide an exhaustive understanding of the problem and the potential solutions that would be impossible as a result of siloed approaches (25). Nonetheless, the OH approach is complex, and there is limited guidance available for investigators regarding the practical design and implementation of OH research.

On the face of it, the prospective gains of the OH approach are largely enshrined in the increasing public health efficiency and cost-effectiveness through a better understanding of disease risk. This can be achieved through shared control and detection efforts. As a result, this will benefit human, animal, and ecosystem health (26). The efforts to identify, systematize, and assess the perceived OH efficiency metrics reveal that standardized evaluations of the One Health approaches are generally lacking (26). The benefits that are widely cited have mainly been premised on modeled projections, rather than outcomes of implemented interventions.

A literature review on this approach further revealed that, out of a pool of over 1,800 unique papers, only seven reported quantitative outcomes. These assessments did not follow the shared methodology and several reviewed only intermediate outcomes. The findings on the One Health approach are largely subjective and the absence of a standardized framework to capture metrics across disciplines could potentially hinder the widespread adoption of One Health among stakeholders (26).

The OH initiative promotes integrated research, surveillance, control programs, and policy frameworks. Considering the transboundary nature of people, pathogens, and ecosystems, ensuring that these international partnerships are built based on these strong foundations is highly important. The vast majority of emerging infectious diseases in humans are zoonotic (27). Often, they escape their natural wildlife reservoirs and infect captive or domestic animals and humans upon cross-species transmission. More often than not, these pathogens spread limitedly among humans; however, once they evolve and transmission has become viable, the effects result in disastrous epidemics, if not pandemics.

The SARS-CoV-2 is an example of novel human pathogens transmitted across borders. This pathogen has had very far-reaching effects on human welfare resulting in a threat to the global community. In light of the above, we must consider the unforeseeable burden that emerging infectious diseases place on global health and the economy. Infectious disease surveillance and pandemic preparedness are essential to mitigate the impact of future threats. Unified global surveillance networks provide unprecedented monitoring data on plant, animal, and human infectious diseases. Using such sources, we can report on current major One Health threats.

The COVID-19 pandemic has established the need for an integrated framework that will grow a strong evidence base to inform decision-making and solution creation. The combined multidisciplinary responses advocated for by the OH approach could potentially do more harm than good; however, it requires that conglomerates and state investment before such a crisis. Surveillance is the key to preparedness. By identifying and monitoring new threats to plant, animal, and human health, early-warning flags can be raised regarding changing epidemiology. This will fast-track pandemic preparedness and response against emerging diseases.

The One Health agenda could potentially be extended to increased international collaborations for drugs and vaccine research and the development of an efficient coronavirus sharing system that includes the DSI of the pathogen. In addition, the OH Agenda can further be promoted by benefit-sharing enforcement across the globe and a review of the technological hindrances between countries that affect and limit fair and equitable sharing.

Based on the preceding discussions in this study, an issue that comes out is the existing gaps in the current ABS mechanism. In addition, this study has briefly highlighted the current and the potential consequences of turning both a deaf ear and a blind eye to the issues at hand. As we continue to tackle COVID-19, perhaps there is a need to take a step back and establish how the ABS/pathogen-sharing process can be streamlined while also taking into account unforeseen circumstances. There is a need to review the overall implementation of this document. Furthermore, there is a need to ask compelling questions that would force the international community to admit that it did not take things into consideration.

There is a need to ponder on the efficiency of the Nagoya Protocol, its implementation, and how this impacts the response to epidemics and pandemics. In addition, the international community must debunk the efficiency of the current pathogen-specific ABS Instruments. Do the pathogen-specific ABS Instruments that are currently in place override or undermine the interests of public health? Finally, is there a way that we can actively ensure compliance to ABS mechanisms as we pursue the journey to COVID-19 treatment and other pathogens?

As we deliberate on the aforementioned, one of the potential solutions to these concerns is the recognition and rapid enforcement of a specialized international instrument for pandemic pathogens under the Nagoya Protocol. The picture that history paints show us the urgency of such an instrument. Moreover, there is a need to develop and implement legislation to support world public health emergencies.

In conclusion, we should consider and explore further fair and equitable worldwide solutions. This could be achieved through transparent open fora, participatory approach, well-defined scope, and governance in consideration of world traditional knowledge and heritage. As Cueni (9) succinctly stated, “Pathogens know no borders, so any obstacle to sharing them and/or their associated information will hinder essential global collaborations with the private and public sectors needed to develop effective countermeasures to disease outbreaks. It is time to question the sense of retaining pathogens within the scope of the Nagoya Protocol and associated national legislation.” It is time for a framework that accommodates and withstands pandemic pressure.

## Author Contributions

SK and CT contributed together to the writing of the article. All authors contributed to the article and approved the submitted version.

## Funding

This research was made under the ABS internship position granted to SK at CTLGH-ILRI and under the supervision of CT, funded in part by the Bill & Melinda Gates Foundation and with UK aid from the UK Foreign, Commonwealth and Development Office?ce (Grant Agreement OPP1127286) under the auspices of the CTLGH. It was also conducted as part of the ILRI-led CGIAR Research Program on Livestock, which is supported by contributors to the CGIAR Trust Fund: https://www.cgiar.org/funders/. Special recognition is made the to ILRI's Environment, Occupational Health, and Safety office (EOHS) and Legal Office Teams. The findings and conclusions contained within are those of the authors and do not necessarily reflect positions or policies of the Bill & Melinda Gates Foundation nor the UK Government.

## Conflict of Interest

The authors declare that the research was conducted in the absence of any commercial or financial relationships that could be construed as a potential conflict of interest.

## Publisher's Note

All claims expressed in this article are solely those of the authors and do not necessarily represent those of their affiliated organizations, or those of the publisher, the editors and the reviewers. Any product that may be evaluated in this article, or claim that may be made by its manufacturer, is not guaranteed or endorsed by the publisher.
